# Supplementary material for: Lysine-36 of Drosophila histone H3.3 supports adult longevity
Source: G3 (Bethesda). 2024 Feb 16;14(4):jkae030. doi: 10.1093/g3journal/jkae030 (PMC10989886; doi:10.1093/g3journal/jkae030)
Supplement: jkae030_Supplementary_Data [file jkae030_supplementary_data.zip › Supplemental_Figures_G3-2024-404848.pdf]

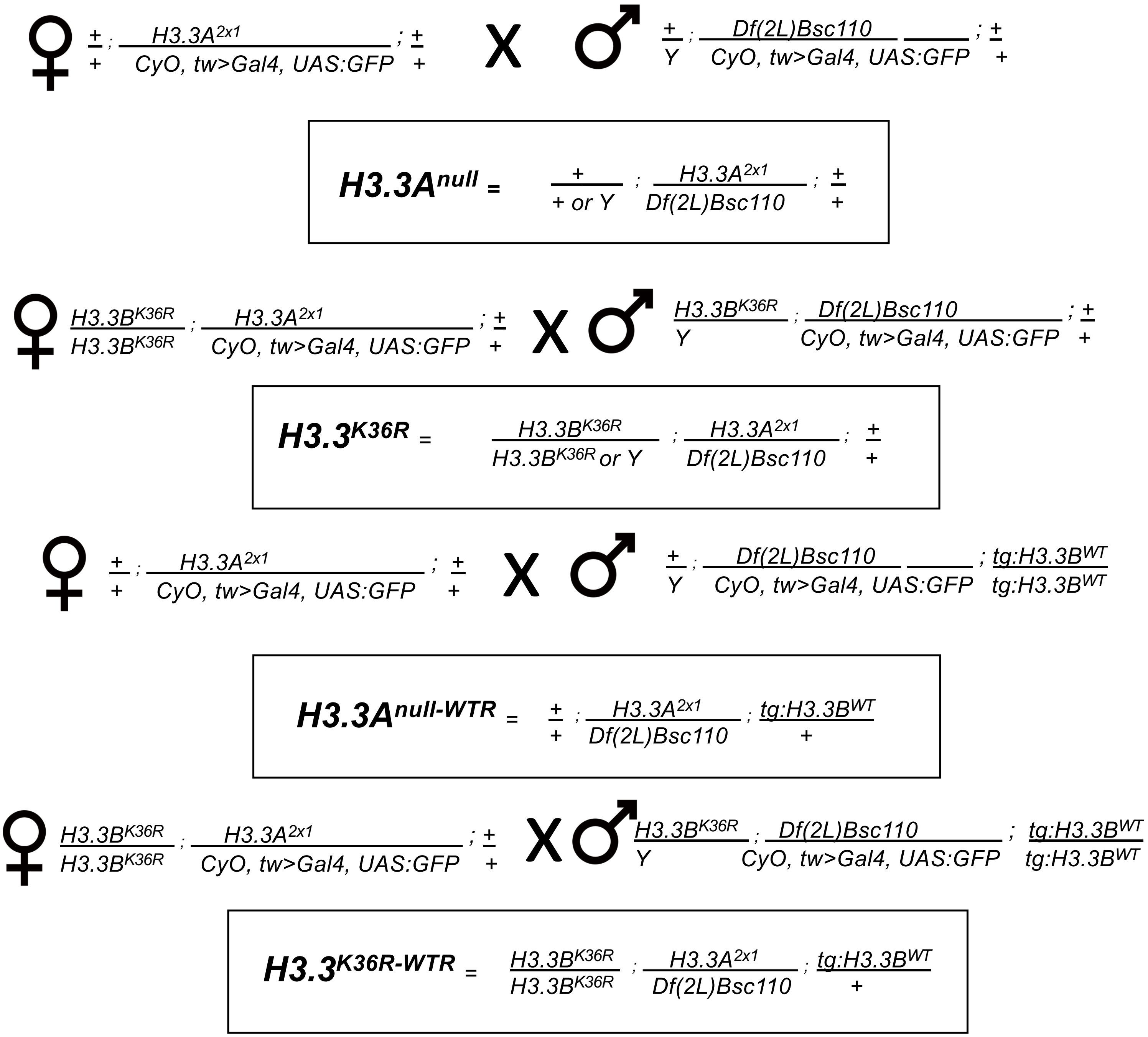

Figure S1. Genetic Crosses Used to Obtain Experimental Genotypes. Genetically related maternal and paternal strains were crossed as above to produce experimental progenty. All X chromosomes carry *y* and w mutations. Second chromosomes with *H3.3A*^{2x1} and *Df(2L)Bsc110* were maintained isogenically, and were identical in all experimental progeny. Progeny were selected for absence of GFP. See Materials and Methods for origins of alleles.

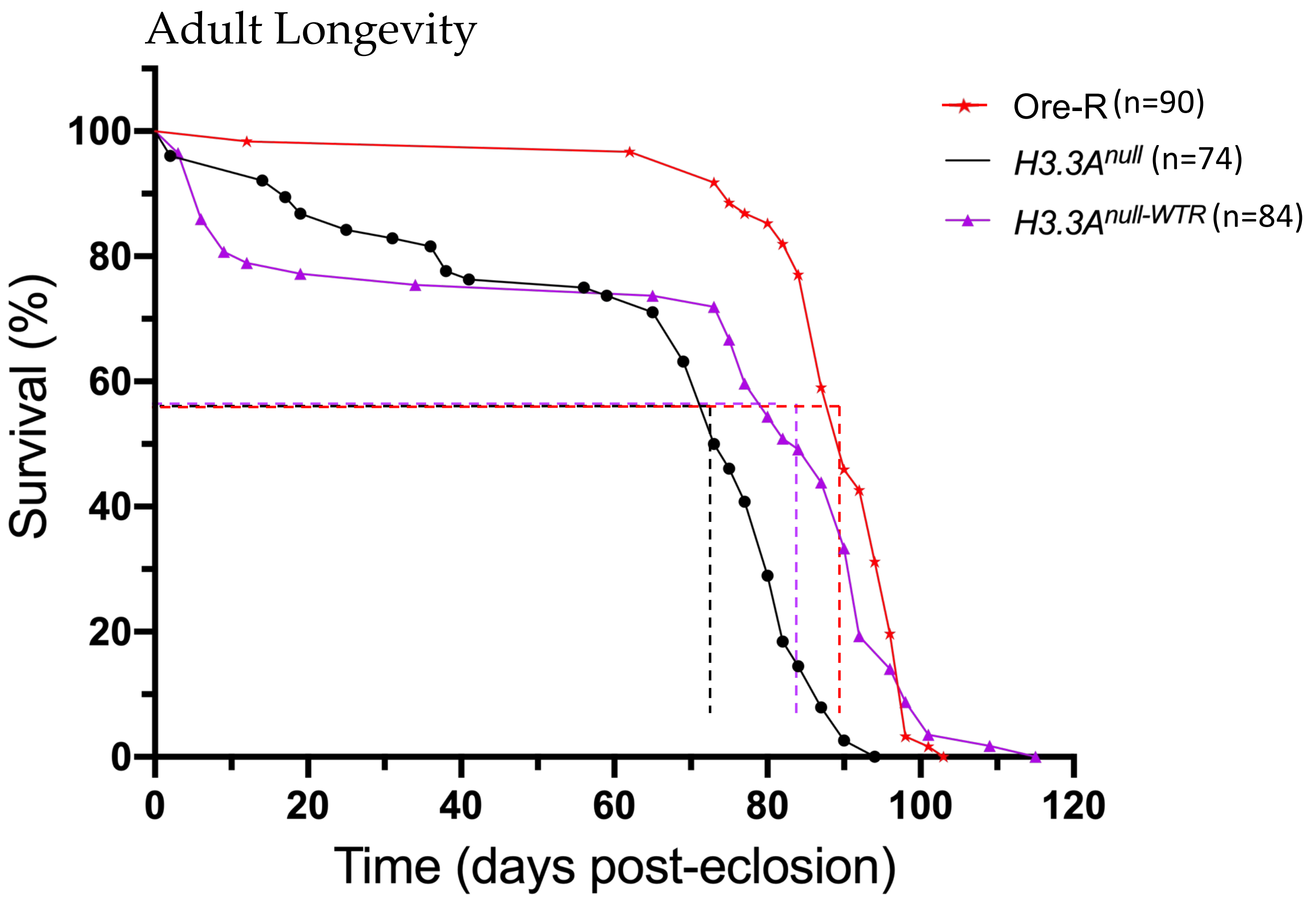

	Ore-R	*H3.3A^null^*	*H3.3A^null-WTR^*
Ore-R	---	$< 10^{-6}$ ****	$< 4.3 \times 10^{-3}$ *
*H3.3A^null^*	$< 10^{-6}$ ****	---	$< 4.2 \times 10^{-3}$ *
*H3.3A^null-WTR^*	$< 4.3 \times 10^{-3}$ *	$< 4.2 \times 10^{-3}$ *	---

Figure S2. *H3.3A^{null}* control animals are slightly less fit than Oregon-R wild type control animals. Adult longevity assays for Oregon-R, *H3.3A^{null}* and *H3.3A$^{null\text{-}WTR}$* flies. Median lifespan was determined (dotted lines) by identifying the day at which 50% of the animals survived. Statistical comparison of survival curves using Gehan-Breslow-Wilcoxon tests are presented in the accompanying table. A Bonferroni correction for multiple comparisons was employed, resulting in the following adjusted significance values: * $p<0.0167$, ** $p<0.0034$, *** $p<3.4 \times 10^{-4}$, ****$p<3.4 \times 10^{-5}$.

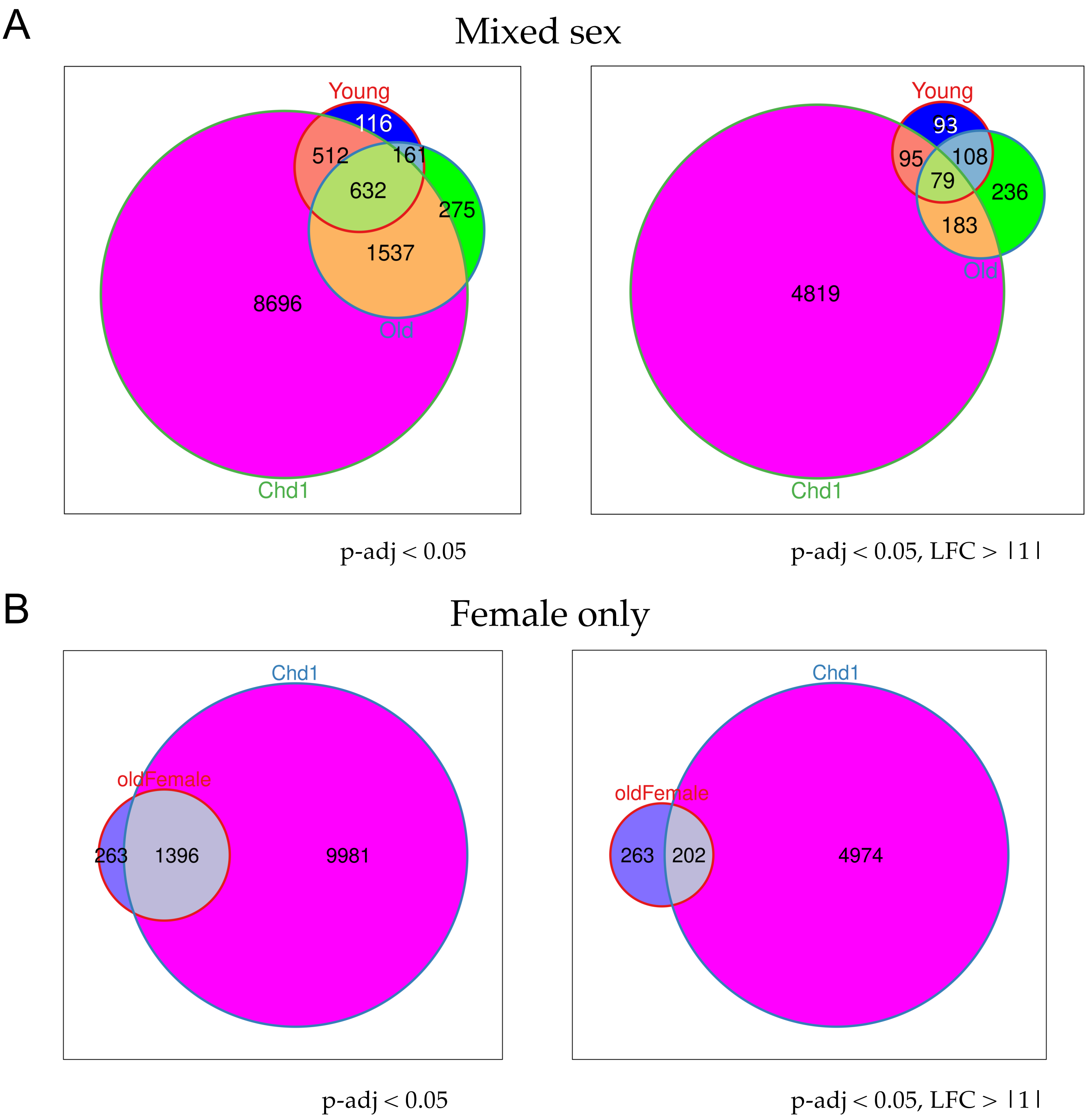

Figure S3. Overlap of DEGs between $H3.3^{K36R}$ and *Chd1* mutants. A) Venn diagrams of Young $H3.3^{K36R}/H3.3A^{null}$, Old $H3.3^{K36R}/H3.3A^{null}$, and *Chd1* / Control DEGs from our Mixed Sex (non-interaction) DESeq2 model (Figure 2) and the *Chd1* data reanalyzed with the same bioinformatic workflow. At left, genes with p-adj < 0.05; at right, with an additional cutoff with LFC > |1|. B) Same as in A, except we performed DESeq2 analysis with sex as a variable, displaying DEGs from females only to more closely match the samples in the *Chd1* study.

Adult Longevity

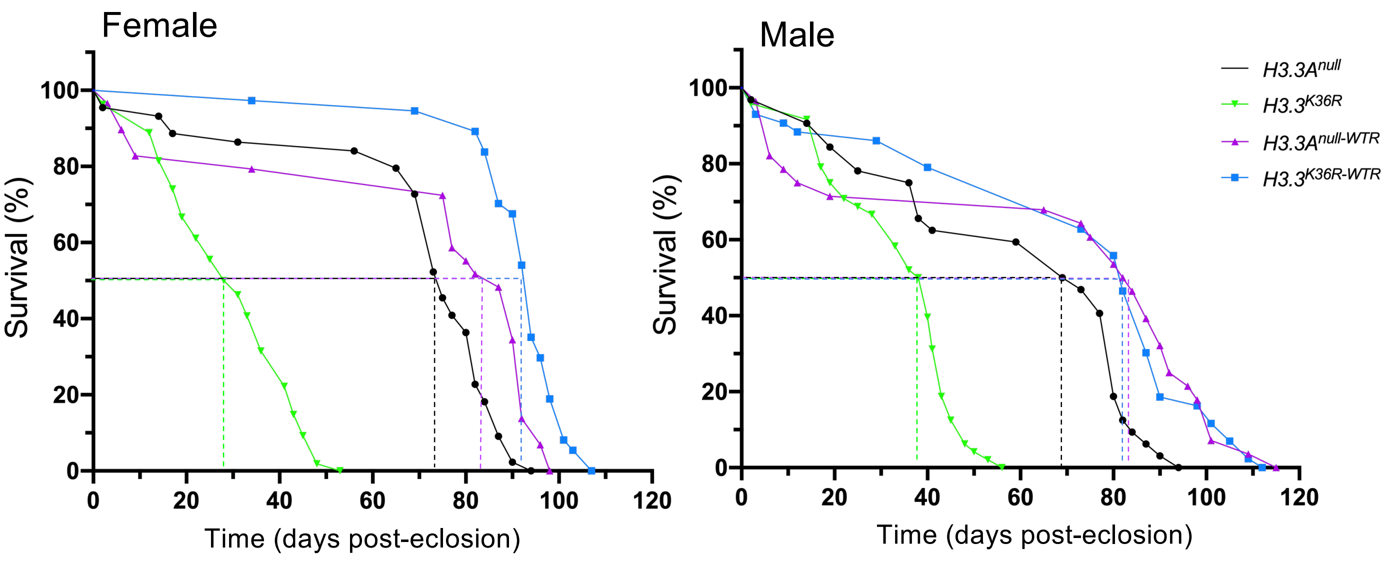

**Female**	$H3.3A^{null}$	$H3.3^{K36R}$	$H3.3A^{null\text{-}WTR}$	$H3.3^{K36R\text{-}WTR}$
$H3.3A^{null}$	---	$< 10^{-6}$<br>****	ns	---
$H3.3^{K36R}$	$< 10^{-6}$<br>****	---	---	$< 10^{-6}$<br>****
$H3.3A^{null\text{-}WTR}$	ns	---	---	$< 2.37 \times 10^{-4}$<br>***
$H3.3^{K36R\text{-}WTR}$	---	$< 10^{-6}$<br>****	$< 2.37 \times 10^{-4}$<br>***	---

**Male**	$H3.3A^{null}$	$H3.3^{K36R}$	$H3.3A^{null\text{-}WTR}$	$H3.3^{K36R\text{-}WTR}$
$H3.3A^{null}$	---	$< 2.37 \times 10^{-4}$<br>***	ns	---
$H3.3^{K36R}$	$< 2.37 \times 10^{-4}$<br>***	---	---	$< 10^{-6}$<br>****
$H3.3A^{null\text{-}WTR}$	ns	---	---	ns
$H3.3^{K36R\text{-}WTR}$	---	$< 10^{-6}$<br>****	ns	---

Figure S4. Adult longevity assays for *H3.3A*^{null} and *H3.3A*$^{null\text{-}WTR}$ controls, and for *H3.3*^{K36R} and *H3.3* $^{K36R\text{-}WTR}$ flies, parsed by sex. Median lifespan was determined (dotted lines) by identifying the day at which 50% of the animals survived. Statistical comparison of survival curves using Gehan-Breslow-Wilcoxon tests are presented in the accompanying table. A Bonferroni correction for multiple comparisons was employed, resulting in the following adjusted significance values: * $p<0.0125$, ** $p<0.0025$, *** $p<2.5 \times 10^{-4}$, ****$p<2.5 \times 10^{-5}$.

A

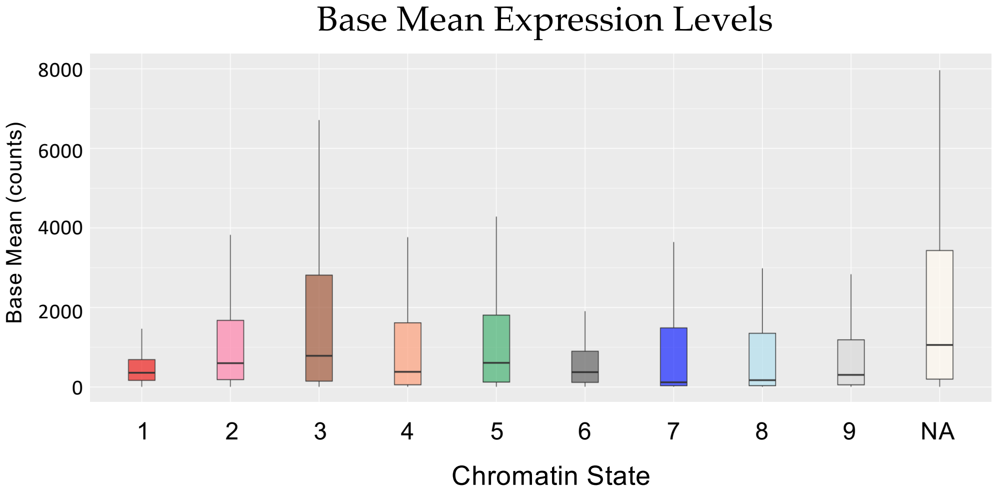

Color	State	Characteristics
	1	H3 K4me2/3, K9ac
	2	H3 K36me3, K79me1
	3	H3 K4me1, K18ac, K27ac
	4	H3K36me1, long introns
	5	H4K16ac
	6	H3K27me2/3
	7	H3K9me2/3 (high)
	8	H3K9me2/3 (mod)
	9	H1, low activity
	NA	< 50% any other state

B

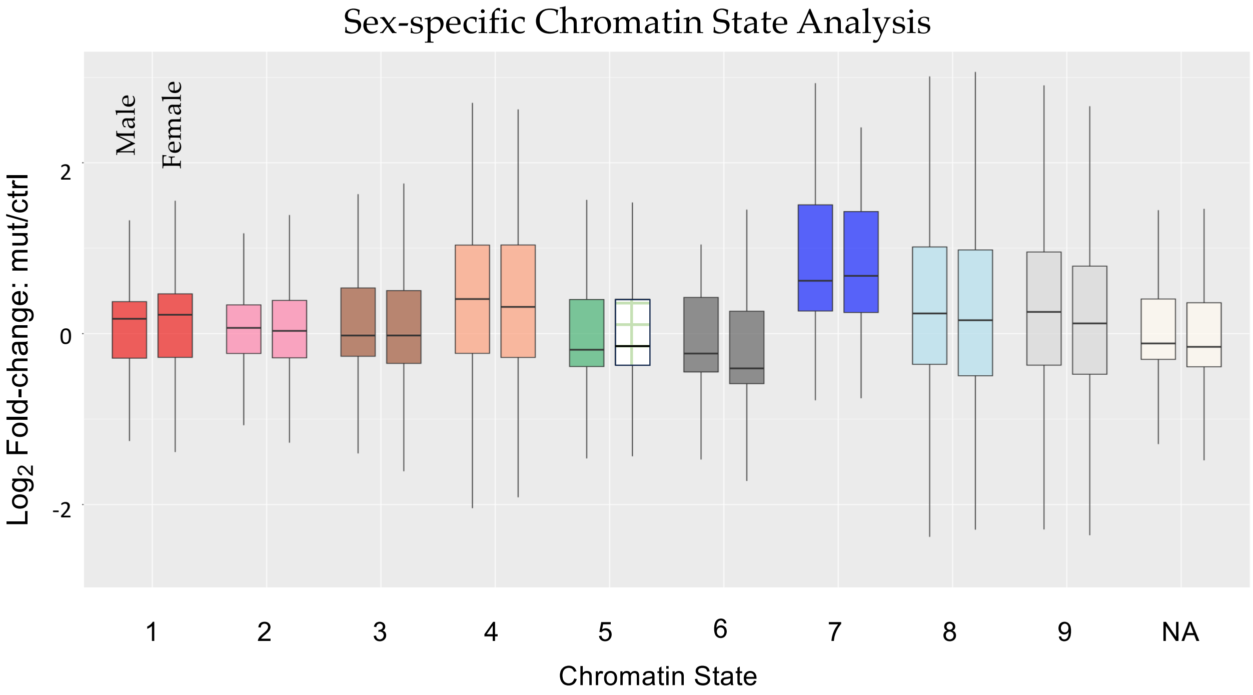

Figure S5. Additional Chromatin State Analyses. **A**) The Base Mean gene expression level for all genotypes in the non-interaction DESeq2 model was plotted for genes in each State and binned as per Figure 5A. **B**) For the set of genes described in Figure 5A, and utilizing a DESeq2 model which considers sex as an additional variable (Fig. S2), separate mut/ctrl Log2 Fold-change values were plotted for Old K36R male and female animals. Because State 5 is specific to male dosage compensation, a cross-hatched pattern was used for the bloxplot of female State 5 DEGs to indicate that the predominant chromatin states of these genes are unknown in females.

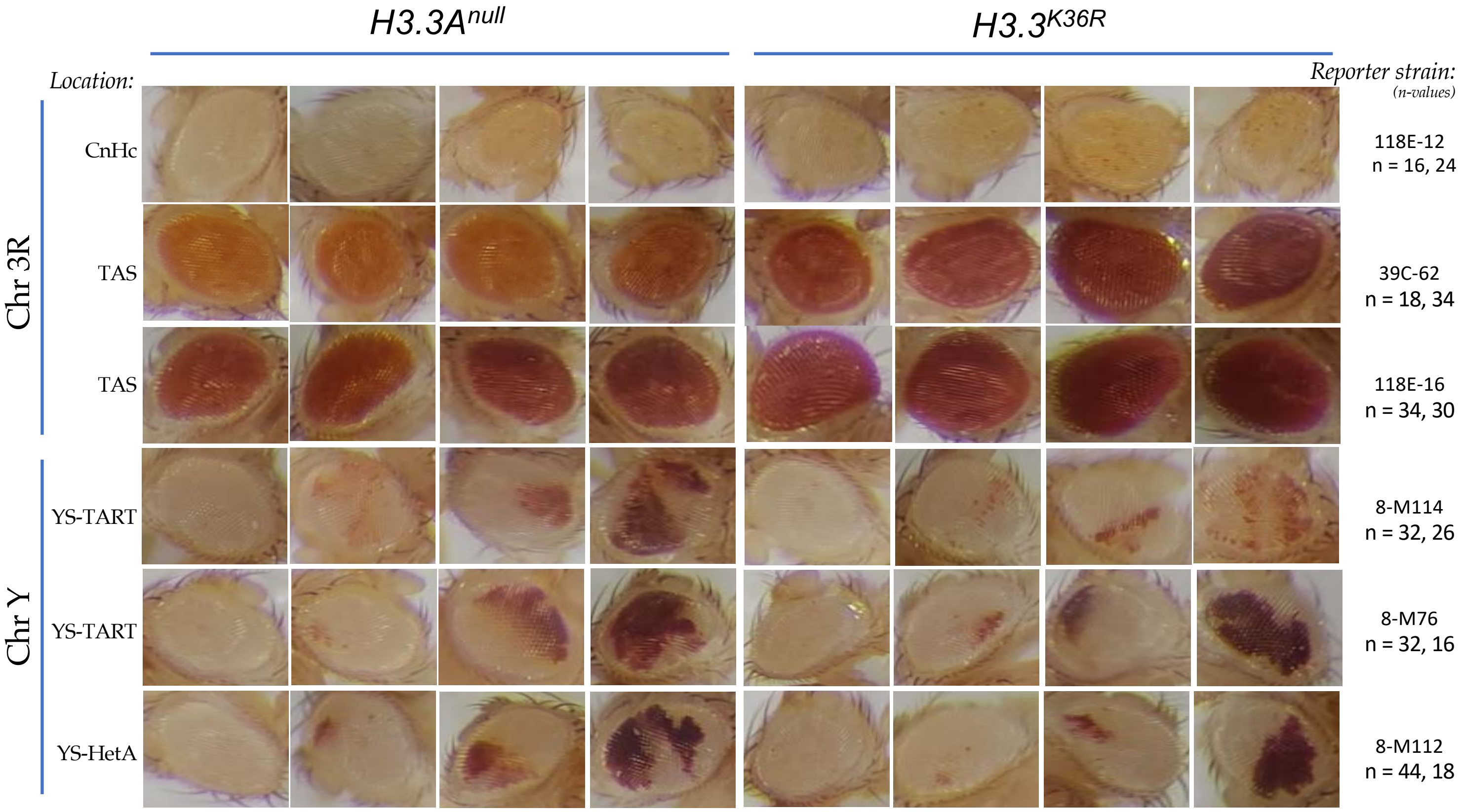

Figure S6. Representative eyes from Position Effect Reporter strains. For each row, four representative eyes for a particular reporter transgene in either the *H3.3A*^{null} control or *H3.3*^{K36R} mutant background are shown. To the left, chromosomal location and type of chromatin region where each reporter insertion is located is indicated. Pericentric heterochromatin (CnHc), Telomere Associated Sequences (TAS), YS-TART (Y chromosome TART), and YS-HetA (Y chromosome-HetA) are abbreviated as indicated. The number (n) of eyes evaluated per *H3.3A*^{null} control (ctrl) and *H3.3*^{K36R} mutant (mut) genotype are indicated at right, below the label for each reporter strain and also listed here: 118E-12 (ctrl=16, mut=24), 39C-62 (ctrl=18, mut=34), 118E-16 (ctrl=30, mut=22), 8-M114 (ctrl=32, mut=26), 8-M76 (ctrl=32, mut=16), 8-M112 (ctrl=44, mut=18).
